# Loop ileostomy-mediated fecal stream diversion is associated with microbial dysbiosis

**DOI:** 10.1080/19490976.2017.1339003

**Published:** 2017-06-16

**Authors:** Emma L. Beamish, Judith Johnson, Elisabeth J. Shaw, Nigel A. Scott, Arnab Bhowmick, Rachael J. Rigby

**Affiliations:** a Division of Biomedical and Life Sciences, Lancaster University, Lancaster, UK; b Lancashire Teaching Hospitals NHS Foundation Trust, Preston, UK

**Keywords:** atrophy, dysbiosis, enteral nutrition, Loop ileostomy, microbiota, morphology, small intestine

## Abstract

Loop ileostomy is an effective procedure to protect downstream intestinal anastomoses. Ileostomy reversal surgery is often performed within 12 months of formation but is associated with substantial morbidity due to severe post-surgical complications. Distal ileum is deprived of enteral nutrition and rendered inactive, often becoming atrophied and fibrotic. This study aimed to investigate the microbial and morphological changes that occur in the defunctioned ileum following loop ileostomy-mediated fecal stream diversion. Functional and defunctioned ileal resection tissue was obtained at the time of loop-ileostomy closure. Intrapatient comparisons, including histological assessment of morphology and epithelial cell proliferation, were performed on paired samples using the functional limb as control. Mucosal-associated microflora was quantified via determination of 16S rRNA gene copy number using qPCR analysis. DGGE with Sanger sequencing and qPCR methods profiled microflora to genus and phylum level, respectively. Reduced villous height and proliferation confirmed atrophy of the defunctioned ileum. DGGE analysis revealed that the microflora within defunctioned ileum is less diverse and convergence between defunctioned microbiota profiles was observed. Candidate Genera, notably Clostridia and Streptococcus, reduced in relative terms in defunctioned ileum. We conclude that Ileostomy-associated nutrient deprivation results in dysbiosis and impaired intestinal renewal in the defunctioned ileum. Altered host-microbial interactions at the mucosal surface likely contribute to the deterioration in homeostasis and thus may underpin numerous postoperative complications. Strategies to sustain the microflora before reanastomosis should be investigated.

## Introduction

Loop ileostomy formation is often performed to reduce septic complications in patients who have undergone extensive bowel surgery. It is most frequently formed following surgical resection in colorectal carcinoma patients to prevent leakage of distal anastomosis, sepsis and the requirement for urgent repeat operation. It functions to protect downstream anastomoses via temporary fecal stream diversion through the abdominal wall. Despite its precautionary therapeutic benefits, the use of fecal stream diversion continues to be debated due to complications associated with ileostomy formation and reversal.

Previous studies have highlighted the effectiveness of a well-formed loop ileostomy at defunctioning downstream intestine following surgery.^1^ Loop ileostomy formation gives rise to 2 contrasting nutritional environments; the proximal ileum remains functional with nutrient and water absorption occurring at the mucosal surface from peristaltic-motioned chyme, while the distal ileum is deprived of luminal contents and thus rendered inactive. A defunctioned loop ileostomy is usually reversed within 12 months, reinstating luminal flow through the entire intestine. In addition to the inconvenience of a second operation for loop closure, the reversal procedure is associated with a substantial morbidity of around 20%.^2-5^ Small bowel obstruction and anastomotic leakage are the most common post-surgical complications with respective incidence rates up to 22% and 10%.^2,6^ Further complications include prolonged postoperative ileus and fecal incontinence, as well as incisional hernia and wound infections at the site of stoma formation.^6-8^ Due to such complications, around 5% of cases prove to be irreversible, leaving patients with a permanent stoma and a reduced quality of life.^9^

Intestinal structure and function are directly related and hence research has thus far focused on the pathophysiology of the defunctioned intestine. Both animal models and human studies have documented the atrophic nature of the defunctioned intestine before closure.^10,11^ In addition, previous research has demonstrated a significant loss of motility and muscle volume in the defunctioned intestine.^11^ Collectively, these physiologic factors are likely to contribute to the post-surgical complications associated with reversal surgery. However, the mechanisms underlying such physiologic changes remain yet to be explored. Clinical studies have reported attempts to stimulate the defunctioned intestine before reanastomosis and loop closure.^12-14^ Thus far, there have been varied reports of success at reducing post-surgical complications, with particular focus placed on postoperative ileus. Such studies were conducted with the aim of gradually activating cellular mechanisms of absorption and motility to restore functionality to the intestine before reversal surgery. However, such rationale and practice vastly disregards the importance of a healthy gut microbiome for proficient intestinal function.

The intestinal microbiota has received considerable interest in recent years and research is beginning to uncover the pivotal role gut microflora play in the complex homeostatic host-microbial interactions essential for maintaining intestinal health (reviewed in ref.^15^). Germ free animal studies revealed substantial defects in the development of various gut-associated lymphoid tissues as well as reduced intestinal epithelial cell (IEC) turnover when compared with specific pathogen free controls.^16,17^ In addition, mice lacking MyD88 adaptor protein, essential for Toll-like receptor signaling, demonstrated that recognition of commensal bacteria by TLR on the apical surface of IEC is fundamental for both maintenance of intestinal homeostasis and repair in response to injury.^18^ The composition of the intestinal microbiota is modulated by various host genetic and environmental factors including medical practices such as antibiotic use, method of childbirth and most significantly, host diet.^19-21^ A plethora of literature now exists detailing the effects of diet modulation on the intestinal microbiota (Reviewed in ref.^22^). Notably, a recent mouse model of total parenteral nutrition (TPN) demonstrated that enteral nutrient deprivation leads to significant shifts in microbial dominance with associated reduced epithelial turnover and intestinal atrophy.^23,24^

We propose that loop ileostomy-mediated fecal stream diversion and the resultant loss of enteral nutrition in the downstream intestine alters microbiota composition, which in turn affects IEC turnover and consequently impacts on intestinal structure and function. This study aimed to investigate microbial and physiologic changes that occur in the defunctioned ileum following fecal stream diversion.

## Materials and methods

### Study inclusion criteria

Research was conducted in accordance with North West Research Ethics Committee guidelines (ref: 13/NW/0695). Patients undergoing loop ileostomy reversal surgery were recruited at Lancashire Teaching Hospitals NHS Trust (Lancashire, UK). Patients who had ongoing bowel pathologies, such as inflammatory bowel disease, or had antibiotic treatments within the last 3 months, were deemed ineligible. Tissue was obtained from 34 patients and all recruited participants had loop ileostomy to protect downstream anastomoses following resection of colorectal cancer tumors. Participant demographics and post-surgical clinical data are presented in Table 1.

**Table 1.** Participant Demographics and Post-surgical Clinical Data.

Participant Demographics and Post-surgical Clinical Data	
Age (years)^*^	58 (± 16)
BMI^*^	26.0 (± 2.4)
Days since ileostomy formation^*^	392 (± 264)
Gender (% females)	51
CRP (mg/L)^*^	87.9 (± 54.8)
Albumin (g/L)^*^	38.1 (± 3.4)
WBC (x10^9^/L)^*^	9.4 (± 1.7)

*Note*.

* The mean is given with standard deviation in parentheses.

### Sample acquisition and handling

**Figure 1.**
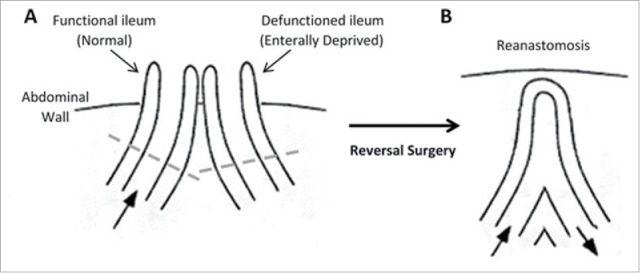
Structure of the intestine. (A) Loop ileostomy and (B) following reanastomosis. Block arrows denote presence and direction of luminal contents flow. Tissue located above dashed lines represent areas of intestine removed before reanastomosis and form the specimen acquired for our research.

Loop ileostomy reversal surgery involves reanastomosis of functional and defunctioned ileum using a linear stapler. A short region of the intestine is routinely removed from the end of each limb before reanastomosis and these are the specimen acquired for this study (see Fig. 1). All specimen were maintained in Minimal Essential Media (Sigma-Aldrich), on ice and were processed within 2 hours of collection. Luminal-associated microflora were pelleted from media. Mucosal-associated microflora were obtained from mucosal biopsies of equal mass from each specimen.

### Histological analyses

Full thickness tissue sections were fixed in 4% paraformaldehyde solution for 24 hours. Morphological analysis was performed on haematoxylin and eosin (H&E) stained sections. Villous height was measured using ImageJ software.^25^ Coded H & E sections were scored for inflammation by a blinded observer. Scoring applied a well-validated system which assigns a score of 0 – 4 for inflammation and mucosal damage based on degree and extent of transmural inflammation, goblet cell depletion, immune infiltrate and architecture distortion.^26^

### Immunofluorescence PCNA analysis

Paraffin sections were dewaxed, rehydrated and subjected to antigen retrieval, by boiling in Sodium Citrate Buffer (10mM Sodium Citrate, 0.05% Tween 20, pH 6.0), using a microwave oven, for 15 minutes. Slides were stained with mouse anti-PCNA (PC10) primary antibody and goat anti-mouse Alexa-Fluor® 488 antibody (ThermoFisher Scientific). Slides were counterstained with Hoescht33342 trihydrochloride nucleic acid stain (Life Technologies) and mounted using VectaShield mounting medium (Vector Laboratories). Number of PCNA-positive IEC per crypt was counted by a blinded observer and the percent of proliferative cells/all nucleated cells was calculated.

### TUNEL assay

TUNEL assay was performed on tissue sections using Click-iT Plus TUNEL Alexa Flour 594 kit, according to manufacturer's protocol (ThermoFisher Scientific). Tissue sections were subjected to antigen retrieval, by boiling in Sodium Citrate Buffer (10mM Sodium Citrate, 0.05% Tween 20, pH 6.0), using a microwave oven, for 15 minutes. Following TUNEL assay, slides were counterstained with Hoescht33342 trihydrochloride nucleic acid stain (Life Technologies) and mounted using VectaShield mounting medium (Vector Laboratories). Total number of apoptotic cells per villous was quantified by a blinded observer.

### Mucosal bacterial DNA extraction

Mucosal biopsies were finely sliced to aid release of associated microflora. Total genomic DNA was extracted using QIAamp Cador pathogen lysis minikit (Qiagen), according to the manufacturer's protocol.

### Luminal bacterial DNA extraction

Media samples were centrifuged at 6000xg to pellet bacteria and resuspended in 500µL buffer ATL (Qiagen). Pellets were subjected to mechanical lysis at maximum speed for 10 minutes in Pathogen lysis tubes (Qiagen), before DNA extraction using QIAamp UCP Pathogen Mini Kit (Qiagen), according to the manufacturer's protocol.

### Denaturation gradient gel electrophoresis (DGGE) profiling

A highly conserved 200bp sequence, neighboring the V3 hypervariable region of the 16S rRNA gene, was amplified using universal 16S rRNA primers, 341F_GC and 518R (Table S1).^27^ Gels were post-stained using SYBR Gold Nucleic Acid Gel Stain and visualized using Image Lab software with 4 seconds exposure. On each DGGE gel, a standard marker, composed of pooled fecal microbial DNA from 5 healthy individuals, was included to allow comparison between gels. DGGE profiles were processed digitally using BioNumerics software (version 7.5; Applied Maths), following the manufacturer's guidelines.

### DGGE band excision and sequencing analysis

Amplicons from DGGE bands of interest were reamplified directly from the gel, via inoculation using a sterile pipette tip. The purity of the PCR products was confirmed via repeat DGGE analysis and extraction until a single band was obtained. Purification was confirmed by running extracted band, neighboring the original sample (Figure S1). Following matched confirmation of target bands with original sample profiles, amplicons were subjected to Sanger sequencing (Source Bioscience), using primers 341F+GC Clamp and 518r. Consensus sequences were generated using BioEdit software (available at www.mbio.ncsu.edu/BioEdit/bioedit.html) and sequences were then classified using nucleotide Basic Local Alignment Search Tool (BLASTn; available online at www.blast.ncbi.nlm.nih.gov.uk). Sequences with complete query coverage and max ident. >99% were classified to genus level. This method of classification was validated first with DNA extracted from known bacterial samples.

### 16SrDNA qRT-PCR

Predominant microbial phyla were compared using phylum-specific and universal 16S rRNA primers (Table S1). 30–50ng of template DNA was added to each PCR reaction. PCR parameters were as follows: 1 cycle of 95°C for 5 min, 35 cycles of 95°C for 20 sec, 61.5°C for 10 sec, annealing, 72°C for 30 sec and 1 cycle of 72°C for 5 min. Data was normalized to total bacteria, using universal primers, and the relative abundances of each phylum determined using the delta delta Ct (2^−∆∆Ct^) algorithm method.^28^

### Determination of total bacterial load

Total bacterial load in the functional and defunctioned intestine was measured via qPCR, using universal eubacterial 16S rRNA primers, Uni334F and Uni514R (Table S1)^29^ and Sybr Green ReadyMix (Sigma-Aldrich). A standard curve constructed for bacterial enumeration using pCR2.1 Topo-TA plasmid vector, containing a cloned portion of the 16S rRNA gene. Bacterial quantification relates to total 16S rRNA gene copy number and not colony forming units or cell counts.

### Statistical analyses

All statistical analyses were performed using SPSS Statistics Desktop (IBM). p ≤ 0.05 was deemed statistically significant (* p ≤ 0.05; ** p ≤ 0.01; *** p ≤ 0.001).

## Results

### Defunctioned intestine is atrophied, but not inflamed

**Figure 2.**
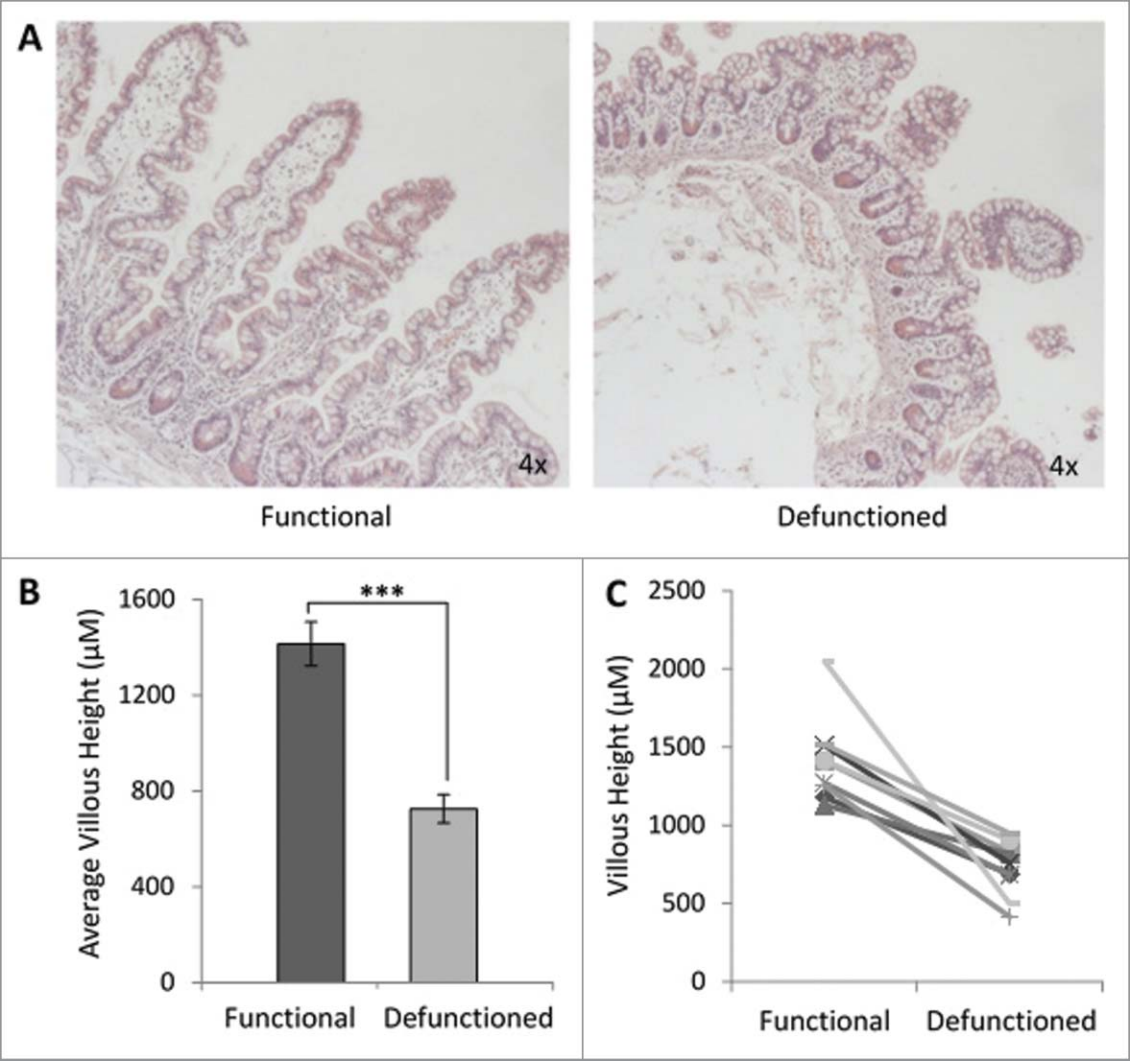
Histological analyses of function and defunctioned ileum. (A) Representative H&E stained sections, magnification 4x. (B) Average villous height ± SEM (n = 9, p = 0.0004) (C) Paired villous height.

We confirmed that intestinal atrophy occurs following loop ileostomy-mediated defunctioning (Fig. 2A). A 47% ± 15% reduction in average villous height was observed in defunctioned ileum of all patients, compared with functional tissue (Fig. 2B). Despite significant atrophy and apparent structural remodeling in defunctioned tissue (Fig. 2A), no significant difference in inflammation was observed compared with functional tissue (Figure S2; n = 9, p ≥ 0.05), suggestive of an absence of chronic inflammation at time of reversal surgery.

### Total bacterial load is reduced in response to defunctioning

**Figure 3.**
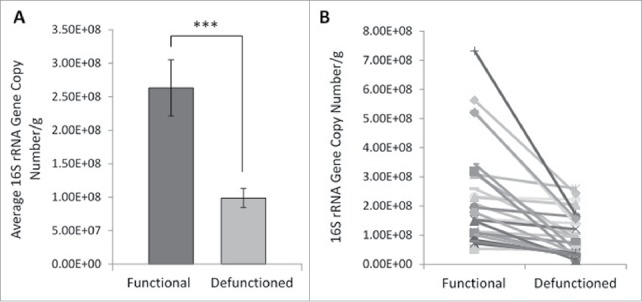
Bacterial enumeration of (A) average- and (B) absolute-16S rRNA gene copy numbers per gram of mucosal tissue in functional and defunctioned intestine (n = 27; p = 0.0003).

Determination of total mucosal bacterial load was performed via quantification of 16S rRNA gene copy number. Small intestinal bacterial load is considered to be around 10ˆ8 cells per ml of luminal content.^30^ In accordance with this, the average mucosa-associated bacterial load in functional ileum was determined to be 2.63.x10ˆ8 gene copies per gram of tissue, compared with 9.89 × 10ˆ7 in the defunctioned (Fig. 3A). This constituted a 62.4% decrease in bacterial load (Fig. 3A; n = 27, p = 0.0003). A reduction occurred consistently across all paired samples tested (Fig. 3B), suggesting that fecal stream diversion and consequential microbial enteral nutrient deprivation leads to a substantial reduction in total bacterial load.

### Loss of microbial diversity and convergence toward a similar bacterial profile occurs in defunctioned intestine

**Figure 4.**
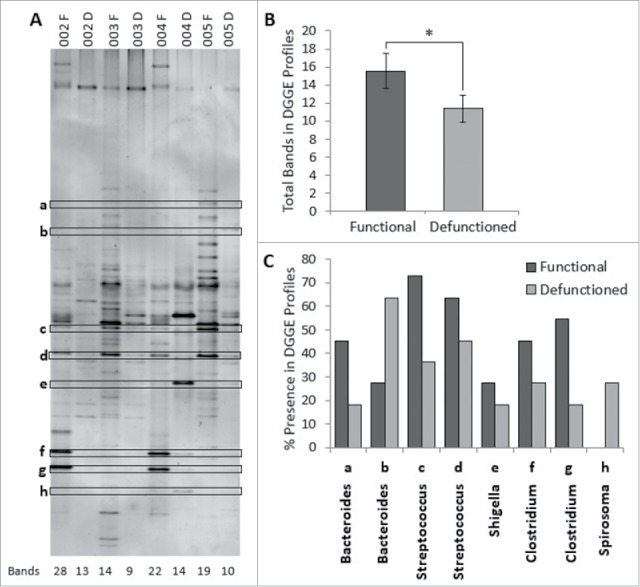
(A) Example of denaturation gradient gel electrophoresis (DGGE) profiles. Band classes are depicted using characters a through h and corresponding bands are enclosed. F, functional ileum; D, defunctioned ileum. (B) Microbial diversity in functional and defunctioned ileum expressed as total number of bands in DGGE profiles (n = 11; P = 0.04). (C) Purified and sequenced band classes expressed as a percentage presence in DGGE profiles across all patients. Characters a-h correspond to the band classes highlighted in A. Inclined, corresponding assigned bacterial genus per band class.

Considering the decrease total bacterial load in defunctioned intestine, microbial composition was investigated using DGGE. Digital processing of DGGE gel image enabled standardized identification of bands across all profiles and generation of a binary presence or absence banding profile for each sample (Fig. 4A). Each band on a DGGE gel represents one or more closely bacterial species therefore the number of bands in each profile reflects the number of different bacterial species and thus overall microbial diversity. On average, the total number of bands in the defunctioned profiles was lower than the number observed in the functional profiles (average of 16 bands to 11 bands, respectively; Fig. 4B), indicating that fecal stream diversion reduces diversity of the intestinal microbiota.

**Figure 5.**
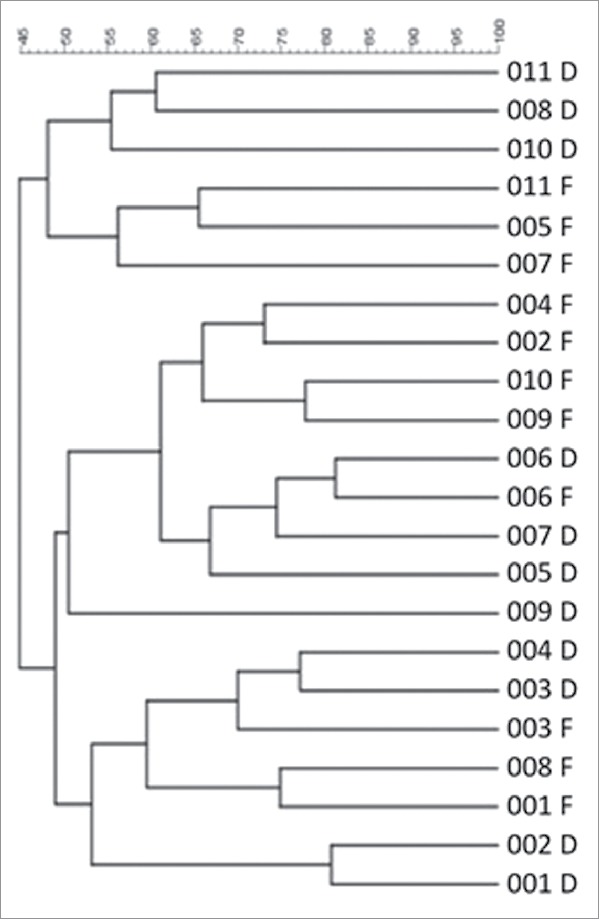
Hierarchical cluster analysis of DGGE profiles represented in graphical form as an UPGMA dendogram. F: functional ileum, D: defunctioned ileum (n = 11).

Hierarchical cluster analysis of binary DGGE banding profiles revealed considerable similarity between the defunctioned profiles, as they cluster together rather than with their paired functional counterparts (Fig. 5). Profiles from functional ileum also clustered together (Fig. 5) demonstrating that, irrespective of interpatient variability, distinct microbial populations exist within the 2 different nutritional environments.

### Dysbiosis is apparent at phylum and genus level in defunctioned bowel

To characterize the apparent disparity in microbial profiles between functional and defunctioned intestine, sequencing analysis was performed on extracted bands classes of interest. In total, 73 distinct band classes were identified across 22 DGGE profiles (11 functional and 11 defunctioned paired samples). A total of 10 band classes, differing in percent presence between functional and defunctioned profiles, were selected and of these, 8 were successfully extracted, purified and sequenced (Fig. S1; Fig. 4C). Subsequent BLAST analysis revealed the highest matched identities from NCBI nucleotide databases for each band class and the highest sequence similarity (99–100%) match was assigned at genus level. Band classes a and b were assigned to the Bacteroides genus, band class c and d to Streptococcus, band class e to Shigella, band class f and g to Clostridium and band class h to Spirosoma (Fig. 4C). Figure 4C also demonstrates percent reductions in Clostridium (18.2% and 36.3% band class f and g, respectively), Shigella/Escherichia (9%, band e) and Streptococcus (36.3% and 18.2%, band c and d) genera across defunctioned, compared with functional profiles. Conversely, an increase in the percentage presence of Spirosoma (27% increase, band h) was observed in the defunctioned profiles. Interestingly, members of the Bacteroides genus were observed to increase or decrease in percent presence across the functional and defunctioned profiles (27.3% reduction and 36% increase, band a and b, respectively; Fig. 4C).

**Figure 6.**
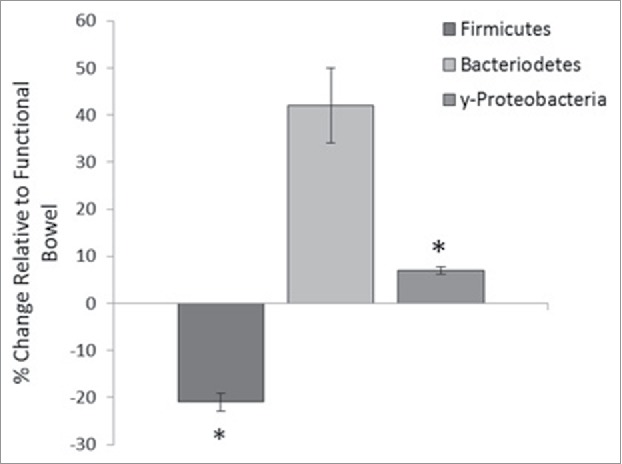
Percentage change of phyla abundance in defunctioned ileum relative to functional. Data normalized to universal 16S rDNA primers (Firmicutes (n = 18, p = 0.02), Bacteroiodetes (n = 18, NS), y-Proteobacteria (n = 9, p ≤ 0.05).

Furthermore, relative quantification of 3 bacterial phyla, Bacteroidetes, Firmicutes and γ-Proteobacteria, was performed on the functional and defunctioned intestine using qRT-PCR. We identified a significant reduction in the relative abundance of the Firmicutes phylum (21% reduction, n = 18; p = 0.02) and a small but significant increase in the γ-Proteobacteria phylum (6.9% increase, n = 9, p = 0.05) in the defunctioned intestine compared with the paired functional controls (Fig. 6). Consistent with DGGE results, the relative abundance of Bacteroidetes varied considerably across our patient cohort (Fig. 6).

### Reduced IEC proliferation was observed in defunctioned crypts

**Figure 7.**
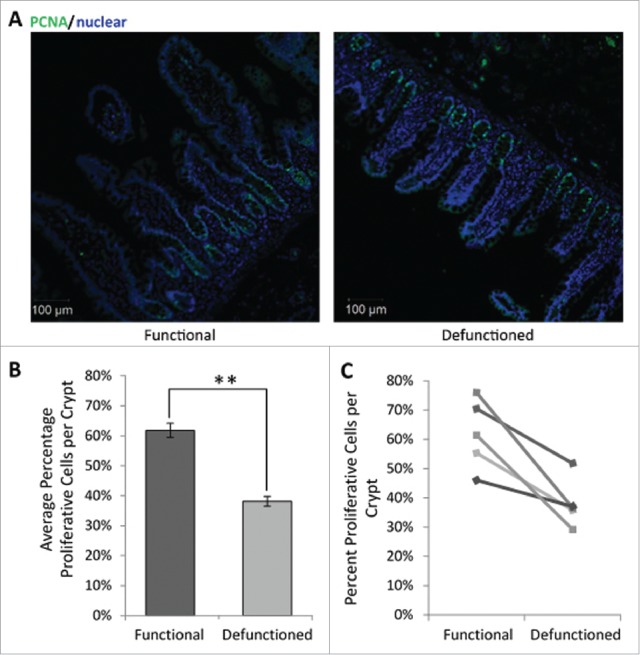
(A) Representative immunofluorescent PCNA labeling (green) to measure proliferation. All nucleated cells, counterstained using Hoechst 33342, are colored blue. Magnification 10x. (B) Average percent proliferating PCNA positive cells/crypt ± SEM (n = 5; p = 0.01). (C) Paired percent PCNA-positive cells per crypt in the functional and defunctioned intestine.

To assess potential functional impact of dysbiosis upon epithelial replenishment, the proportion of proliferative cells per crypt were determined. Loop ileostomy-mediated defunctioning resulted in a decline in the percent of PCNA-positive IECs per crypt (38.1% ± 8.3%; Fig. 7) compared with that observed in the functional controls (61.8% ± 11.9%; Fig. 7). This represents an overall average reduction of 23.7% ± 4% (n = 5, p = 0.01), supporting evidence that microbial changes influence IEC proliferation. Furthermore, TUNEL staining revealed no difference in rates of apoptosis between the functional and defunctioned ileum (data not shown). This suggests that reduction in villous height is due to decreased proliferation of IEC rather than increased apoptosis, which may be due to the lack of pro-proliferative microbial signals.

## Discussion

Our study has demonstrated a strong relationship between loop ileostomy-mediated fecal stream diversion and profound alterations in the intestinal microbiota, termed dysbiosis. This may be a causative factor in the observed mucosal atrophy and consequent post-surgical complications. These results provide novel insights into the effect of loop ileostomy-associated enteral nutrient deprivation on the intestinal microbiota, which may underpin the substantial morbidity rate observed following loop ileostomy closure.

The data presented demonstrates substantial distortion of intestinal mucosal architecture following fecal stream diversion, with significant atrophy of villi in the defunctioned limb. This is consistent with findings reported in previous animal and human studies.^10,11^ Furthermore, a comprehensive morphological study of the defunctioned ileum reported several additional physiologic changes, including loss of smooth muscle area and reduced isometric contractility.^11^ Such physiologic consequences of loop ileostomy-mediated defunctioning, have previously been suggested to occur as a direct result of cellular nutrient deprivation.^31,32^ As a result, present clinical studies aiming to reduce post-surgical complications following loop ileostomy reversal have administered a ‘feed’ to the defunctioned ileum before surgery. The feed is composed of a saline solution and thickening agent which is used to improve cellular functionality before reinstating luminal flow, but offers no nutritional value.^14^ This study demonstrates for the first time that loop ileostomy leads to microbial dysbiosis at both the phylum and genus level. In contrast to mucosal cells, which receive blood-borne nutrition, gut microbes in the defunctioned ileum are nutritionally deprived. Considering the microflora, the feed used in Abrisqueta *et al*. 2014 provides no nutritional sustenance and is therefore unlikely to restore or support the patient's microbiome before reversal surgery.^14^

Profound dysbiosis was observed in the defunctioned ileum at the time of reversal. We report a significant reduction in total bacterial load in the defunctioned intestine, as determined by a reduction in 16S rRNA gene copy number, as well as broad shifts in microbiota composition between functional and defunctioned intestine. Of particular note, we found a significant loss of the predominant phylum Firmicutes, reported to be decreased in IBD and associated with obesity.^33,34^ A concomitant increase in γ-Proteobacteria is observed in the defunctioned limb. Increases in this phylum have also been described in IBD.^35^ Similar dysbiotic changes were observed in a study investigating the effects of TPN on the microbiota of human small intestine.^36^ Interestingly, the study reports a correlation between duration of TPN use and increased severity of dysbiosis; participants fed with TPN either partially, or for less than 6 weeks, somewhat maintained microbial diversity compared with one participant given TPN for >2 months. Given that the downstream intestine in participants of this study remained defunctioned for an average of one year, it is unsurprising that we observed such profound dysbiosis.

The influence of the gut microbiota upon intestinal function should not be underestimated as the importance of both bacterial derived nutrients and direct microbial stimulation for maintaining intestinal replenishment and consequently health is well documented. For example, bacterial derived butyrate is a primary energy source for IECs and recognition of commensal microbes by epithelial TLR is known to be crucial for maintaining intestinal homeostasis.^18,37^ Intestinal dysbiosis has been linked to the pathogenesis of numerous chronic diseases due to the induction of a proinflammatory state within the intestine.^38-40^ Interestingly, we identified that there was no significant inflammation observed in the defunctioned ileum at the time of loop ileostomy reversal. We consider this finding linked to the reduction observed in total bacterial load following nutrient deprivation. The intestinal microbiome is sensitive to local nutritional fluctuations and extreme prolonged starvation in the defunctioned ileum appears to rapidly deplete bacterial load. It is likely that an initial period of inflammation following ileostomy formation occurs, as evidenced by the fibrotic nature of intestinal tissue. Subsequently a state of ‘dysbiotic equilibrium’ is reached and mucosal homeostasis, be it at a compromised capacity, is reinstated preventing chronic inflammation. However, reanastomosis to reinstate luminal flow through the defunctioned intestine could restore bacterial load while maintaining dysbiosis therefore putting patients at risk of complications.

Blood tests, measuring CRP, Albumin and WBC are routinely used as predictors of post-surgical complications and clinical outcomes.^41,42^ We report a substantial elevation in post-surgical CRP levels with a concomitant reduction in serum albumin in our patient cohort. Serum albumin levels decrease during an inflammatory response to surgery or infection as the liver shifts its production toward acute phase proteins. Elevated CRP is a natural part of the inflammatory response to surgery, but may also be raised in infection, and can be an indicator of anastomotic leak. Elevated CRP is possibly due to incompetent barrier function of the defunctioned bowel during the period following reanastomosis. In addition to mucosal atrophy, depletion of the microbiome is associated with altered intestinal permeability and activation of local immune/stromal cells.^43^ It is plausible that dysbiosis-driven changes in the defunctioned limb following reversal surgery, contribute to an increased risk of developing post-surgical complications, such as small bowel obstruction or ileus. This should be investigated further to evaluate future clinical applications, for example through provision of nutritional support to the defunctioned limb. Patients did not receive any ‘bowel preparation’ before ileostomy reversal; such preparations may influence, particularly luminal, microbiota.

We have also demonstrated that ileostomy-associated defunctioning results in a reduction in IEC proliferation. Mice fed exclusively on TPN also demonstrated a reduction in IEC proliferation in a MyD88 dependent manner.^23^ MyD88 knockout (^−/−^) mice presented normal levels of IEC proliferation irrespective of enteral nutrient deprivation, but wild-type TPN mice demonstrated a characteristic significant shift in microbial dominance from Firmicutes to Proteobacteria, consistent with our present findings.^23^ Furthermore, physiologic preservation of epithelial barrier function in MyD88 ^−/−^ TPN fed mice was also reported, indicating minimal disruption to intestinal morphology, suggesting that host immunity has an impact upon the microflora composition.^23^ Collectively, it is reasonable to conclude that the reduction we observed in IEC proliferation and resultant intestinal atrophy is highly likely to be a consequence of dysbiosis, via altered host-microbial interactions at the intestinal surface, rather than due to nutrient deprivation exclusively.

Collectively, these findings could support future feasibility studies to provide the defunctioned bowel with nutritional sustenance before reanastomosis, supporting microbiome restoration. Nutritional support, in the form of elemental diet, could be supplemented with prebiotics or fecal effluent from the functioning limb.

## Conclusion

Loop ileostomy reversal surgery is associated with substantial morbidity and post-surgical complications. We have shown that loop ileostomy-associated fecal stream diversion results in intestinal dysbiosis and likely influences the development of impaired intestinal function. We propose that novel therapeutics should focus on restoring the microflora to health to promote intestinal functionality and potentially further reduce postoperative complications.

## Supplementary Material

KGMI_A_1339003_Supplemental.docx

## References

[1] Winslet MC, Drolc Z, Allan A, Keighley MR Assessment of the defunctioning efficiency of the loop ileostomy. Dis Colon Rectum 1991; 34(8):699-703; PMID:1855427; https://doi.org/10.1007/BF02050354 1855427

[2] Pemberton JH, Kelly KA, Beart RW Jr, Dozois RR, Wolff BG, Ilstrup DM Ileal pouch-anal anastomosis for chronic ulcerative colitis. Long-term results. Ann Surg 1987; 206(4):504-13; PMID:3662660; https://doi.org/10.1097/00000658-198710000-00011 3662660 PMC1493214

[3] Chow A, Tilney HS, Paraskeva P, Jeyarajah S, Zacharakis E, Purkayastha S The morbidity surrounding reversal of defunctioning Ileostomies: A systematic review of 48 studies including 6,107 cases. Int J Colorectal Dis 2009; 24(6):711-23; PMID:19221766; https://doi.org/10.1007/s00384-009-0660-z 19221766

[4] van Westreenen HL, Visser A, Tanis PJ, Bemelman WA Morbidity related to defunctioning Ileostomy closure after Ileal Pouch-Anal anastomosis and low colonic anastomosis. Int J Colorectal Dis 2012; 27(1):49-54; PMID:21761119; https://doi.org/10.1007/s00384-011-1276-7 21761119 PMC3249166

[5] El-Hussuna A, Lauritsen M, Bulow S Relatively High Incidence of complications after loop Ileostomy reversal. Dan Med J 2012; 59(10):A4517 23158893

[6] Musters GD, Atema JJ, van Westreenen HL, Buskens CJ, Bemelman WA, Tanis PJ Ileostomy closure by colorectal surgeons results in less major morbidity: Results from an institutional change in practice and awareness. Int J Colorectal Dis 2016; 31(3):661-7; PMID:26732261; https://doi.org/10.1007/s00384-015-2478-1 26732261 PMC4773497

[7] D'Haeninck A, Wolthuis AM, Penninckx F, D'Hondt M, D'Hoore A Morbidity after closure of a defunctioning loop Ileostomy. Acta Chir Belg 2011; 111(3):136-41; PMID:21780519; https://doi.org/10.1080/00015458.2011.11680724 21780519

[8] Hasegawa H, Radley S, Morton DG, Keighley MR Stapled versus sutured closure of loop Ileostomy: A randomized controlled trial. Ann Surg 2000; 231(2):202-4; PMID:10674611; https://doi.org/10.1097/00000658-200002000-00008 10674611 PMC1420987

[9] Bailey CM, Wheeler JM, Birks M, Farouk R The incidence and causes of permanent stoma after anterior resection. Colorectal Dis 2003; 5(4):331-4; PMID:12814411; https://doi.org/10.1046/j.1463-1318.4.s1.1_78.x 12814411

[10] Ekelund KM, Ekblad E Structural, neuronal, and functional adaptive changes in atrophic rat Ileum. Gut 1999; 45(2):236-45; PMID:10403736; https://doi.org/10.1136/gut.45.2.236 10403736 PMC1727608

[11] Williams L, Armstrong MJ, Finan P, Sagar P, Burke D The effect of faecal diversion on human Ileum. Gut 2007; 56(6):796-801; PMID:17229794; https://doi.org/10.1136/gut.2006.102046 17229794 PMC1954877

[12] Miedema BW, Kohler L, Smith CD, Phillips SF, Kelly KA Preoperative perfusion of bypassed Ileum does not improve postoperative function. Dig Dis Sci 1998; 43(2):429-35; PMID:9512141; https://doi.org/10.1023/A:1018887212921 9512141

[13] Abrisqueta J, Abellan I, Frutos MD, Lujan J, Parrilla P [Afferent loop stimulation prior to ileostomy closure]. Cir Esp 2013; 91(1):50-2; PMID:23153779; https://doi.org/10.1016/j.ciresp.2012.09.002 23153779

[14] Abrisqueta J, Abellan I, Lujan J, Hernandez Q, Parrilla P Stimulation of the efferent limb before ileostomy closure: A randomized clinical trial. Dis Colon Rectum 2014; 57(12):1391-6; PMID:25380005; https://doi.org/10.1097/DCR.0000000000000237 25380005

[15] Round JL, Mazmanian SK The gut microbiota shapes intestinal immune responses during health and disease. Nat Rev Immunol 2009; 9(5):313-23; PMID:19343057; https://doi.org/10.1038/nri2515 19343057 PMC4095778

[16] Perey DY, Good RA Experimental arrest and induction of lymphoid development in intestinal lymphoepithelial tissues of rabbits. Lab Invest 1968; 18(1):15-26; PMID:4966916 4966916

[17] Savage DC, Siegel JE, Snellen JE, Whitt DD Transit Time of epithelial cells in the small intestines of germfree mice and Ex-germfree mice associated with indigenous microorganisms. Appl Environ Microbiol 1981; 42(6):996-1001; PMID:7198427 7198427 10.1128/aem.42.6.996-1001.1981 PMC244145

[18] Rakoff-Nahoum S, Paglino J, Eslami-Varzaneh F, Edberg S, Medzhitov R Recognition of commensal microflora by toll-like receptors is required for intestinal homeostasis. Cell 2004; 118(2):229-41; PMID:15260992; https://doi.org/10.1016/j.cell.2004.07.002 15260992

[19] De La Cochetiere MF, Durand T, Lepage P, Bourreille A, Galmiche JP, Dore J Resilience of the dominant human fecal microbiota upon short-course antibiotic challenge. J Clin Microbiol 2005; 43(11):5588-92 16272491 10.1128/JCM.43.11.5588-5592.2005 PMC1287787

[20] Gronlund MM, Lehtonen OP, Eerola E, Kero P Fecal microflora in healthy infants born by different methods of delivery: permanent changes in intestinal flora after cesarean delivery. J Pediatr Gastroenterol Nutr 1999; 28(1):19-25; PMID:9890463; https://doi.org/10.1097/00005176-199901000-00007 9890463

[21] De Filippo C, Cavalieri D, Di Paola M, Ramazzotti M, Poullet JB, Massart S, Collini S, Pieraccini G, Lionetti P Impact of Diet in shaping gut microbiota revealed by a comparative study in children from europe and rural Africa. Proc Natl Acad Sci U S A 2010; 107(33):14691-6; PMID:20679230; https://doi.org/10.1073/pnas.1005963107 20679230 PMC2930426

[22] Graf D, Di Cagno R, Fak F, Flint HJ, Nyman M, Saarela M, Watzl B Contribution of diet to the composition of the human gut microbiota. Microb Ecol Health Dis 2015; 26:26164; PMID:25656825 25656825 10.3402/mehd.v26.26164 PMC4318938

[23] Miyasaka EA, Feng Y, Poroyko V, Falkowski NR, Erb-Downward J, Gillilland MG 3rd, Mason KL, Huffnagle GB, Teitelbaum DH Total parenteral nutrition-associated lamina propria inflammation in mice is mediated by a Myd88-dependent mechanism. J Immunol 2013; 190(12):6607-15; PMID:23667106; https://doi.org/10.4049/jimmunol.1201746 23667106 PMC3679213

[24] Feng Y, Teitelbaum DH epidermal growth factor/Tnf-Alpha transactivation modulates epithelial cell proliferation and apoptosis in a mouse model of parenteral nutrition. Am J Physiol Gastrointest Liver Physiol 2012; 302(2):G236-49; PMID:22075779; https://doi.org/10.1152/ajpgi.00142.2011 22075779 PMC3341111

[25] Schneider CA, Rasband WS, Eliceiri KW Nih image to Imagej: 25 Years of image analysis. Nat Methods 2012; 9(7):671-5; PMID:22930834; https://doi.org/10.1038/nmeth.2089 22930834 PMC5554542

[26] Rath HC, Herfarth HH, Ikeda JS, Grenther WB, Hamm TE Jr, Balish E, Taurog JD, Hammer RE, Wilson KH, et al. Normal luminal bacteria, especially bacteroides species, mediate chronic colitis, gastritis, and arthritis in Hla-B27/Human Beta2 microglobulin transgenic rats. J Clin Invest 1996; 98(4):945-53; PMID:8770866; https://doi.org/10.1172/JCI118878 8770866 PMC507509

[27] Muyzer G, de Waal EC, Uitterlinden AG Profiling of complex microbial populations by denaturing gradient gel electrophoresis analysis of polymerase chain reaction-amplified genes coding for 16s Rrna. Appl Environ Microbiol 1993; 59(3):695-700; PMID:7683183 7683183 10.1128/aem.59.3.695-700.1993 PMC202176

[28] Livak KJ, Schmittgen TD Analysis of relative gene expression data using real-time quantitative Pcr and the 2(-Delta Delta C(T)) method. Methods 2001; 25(4):402-8; https://doi.org/10.1006/meth.2001.1262 11846609

[29] Hartman AL, Lough DM, Barupal DK, Fiehn O, Fishbein T, Zasloff M, Eisen JA Human gut microbiome adopts an alternative state following small bowel transplantation. Proc Natl Acad Sci U S A 2009; 106(40):17187-92; PMID:19805153; https://doi.org/10.1073/pnas.0904847106 19805153 PMC2746123

[30] Berg RD The indigenous gastrointestinal microflora. Trends Microbiol 1996; 4(11):430-5; PMID:8950812; https://doi.org/10.1016/0966-842X(96)10057-3 8950812

[31] Altmann GG Influence of bile and pancreatic secretions on the size of the intestinal villi in the Rat. Am J Anat 1971; 132(2):167-77; PMID:5112467; https://doi.org/10.1002/aja.1001320204 5112467

[32] Keren DF, Elliott HL, Brown GD, Yardley JH Atrophy of villi with hypertrophy and hyperplasia of paneth cells in isolated (Thiry-Vella) Ileal loops in Rabbits. light-microscopic studies. Gastroenterology 1975; 68(1):83-93 1116668

[33] Sokol H, Pigneur B, Watterlot L, Lakhdari O, Bermudez-Humaran LG, Gratadoux JJ, Blugeon S, Bridonneau C, Furet JP, et al. Faecalibacterium prausnitzii is an anti-inflammatory commensal bacterium identified by gut microbiota analysis of crohn disease patients. Proc Natl Acad Sci U S A 2008; 105(43):16731-6; PMID:18936492; https://doi.org/10.1073/pnas.0804812105 18936492 PMC2575488

[34] Backhed F, Ding H, Wang T, Hooper LV, Koh GY, Nagy A, Semenkovich CF, Gordon JI The gut microbiota as an environmental factor that regulates fat storage. Proc Natl Acad Sci U S A 2004; 101(44):15718-23; PMID:15505215; https://doi.org/10.1073/pnas.0407076101 15505215 PMC524219

[35] Baumgart M, Dogan B, Rishniw M, Weitzman G, Bosworth B, Yantiss R, Orsi RH, Wiedmann M, McDonough P, et al. Culture independent analysis of Ileal mucosa reveals a selective increase in invasive escherichia coli of novel phylogeny relative to depletion of clostridiales in crohn's disease involving the Ileum. Isme J 2007; 1(5):403-18; PMID:18043660; https://doi.org/10.1038/ismej.2007.52 18043660

[36] Ralls MW, Miyasaka E, Teitelbaum DH Intestinal microbial diversity and perioperative complications. JPEN J Parenter Enteral Nutr 2014; 38(3):392-9; PMID:23636012; https://doi.org/10.1177/0148607113486482 23636012 PMC4183124

[37] Csordas A Butyrate, aspirin and colorectal cancer. Eur J Cancer Prev 1996; 5(4):221-31; PMID:8894559; https://doi.org/10.1097/00008469-199608000-00002 8894559

[38] Walker AW, Sanderson JD, Churcher C, Parkes GC, Hudspith BN, Rayment N, Brostoff J, Parkhill J, Dougan G, et al. High-throughput clone library analysis of the mucosa-associated microbiota reveals dysbiosis and differences between inflamed and non-inflamed regions of the intestine in inflammatory bowel disease. BMC Microbiol 2011; 11:7; PMID:21219646; https://doi.org/10.1186/1471-2180-11-7 21219646 PMC3032643

[39] Sobhani I, Tap J, Roudot-Thoraval F, Roperch JP, Letulle S, Langella P, Corthier G, Tran Van Nhieu J, Furet JP Microbial dysbiosis in colorectal cancer (Crc) patients. PLoS One 2011; 6(1):e16393; PMID:21297998; https://doi.org/10.1371/journal.pone.0016393 21297998 PMC3029306

[40] Jiang W, Wu N, Wang X, Chi Y, Zhang Y, Qiu X, Hu Y, Li J, Liu Y Dysbiosis gut microbiota associated with inflammation and impaired mucosal immune function in intestine of humans with non-alcoholic fatty liver disease. Sci Rep 2015; 5:8096; PMID:25644696; https://doi.org/10.1038/srep08096 25644696 PMC4314632

[41] Ortega-Deballon P, Radais F, Facy O, d'Athis P, Masson D, Charles PE, Cheynel N, Favre JP, Rat P C-Reactive Protein Is an Early Predictor of Septic Complications after Elective Colorectal Surgery. World J Surg 2010; 34(4):808-14; PMID:20049435; https://doi.org/10.1007/s00268-009-0367-x 20049435 PMC2877195

[42] Hubner M, Mantziari S, Demartines N, Pralong F, Coti-Bertrand P, Schafer M Postoperative albumin drop is a marker for surgical stress and a predictor for clinical outcome: A pilot study. Gastroenterol Res Pract 2016; 2016:8743187; PMID:26880899; https://doi.org/10.1155/2016/8743187 26880899 PMC4736779

[43] Cani PD, Possemiers S, Van de Wiele T, Guiot Y, Everard A, Rottier O, Geurts L, Naslain D, Neyrinck A, et al. Changes in gut microbiota control inflammation in obese mice through a mechanism involving Glp-2-driven improvement of gut permeability. Gut 2009; 58(8):1091-103; PMID:19240062; https://doi.org/10.1136/gut.2008.165886 19240062 PMC2702831

