# Supplementary material for: Loop ileostomy-mediated fecal stream diversion is associated with microbial dysbiosis
Source: Gut Microbes. 2017 Jun 16;8(5):467–78. doi: 10.1080/19490976.2017.1339003 (PMC5628638; doi:10.1080/19490976.2017.1339003)
Supplement: KGMI_A_1339003_Supplemental.docx [file kgmi-08-05-1339003-s001.docx]

Supplementary Information

Table S1 – Bacterial 16S rRNA PCR primers


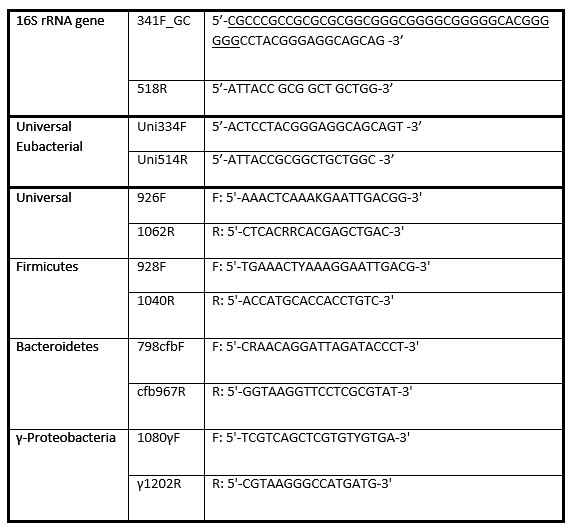


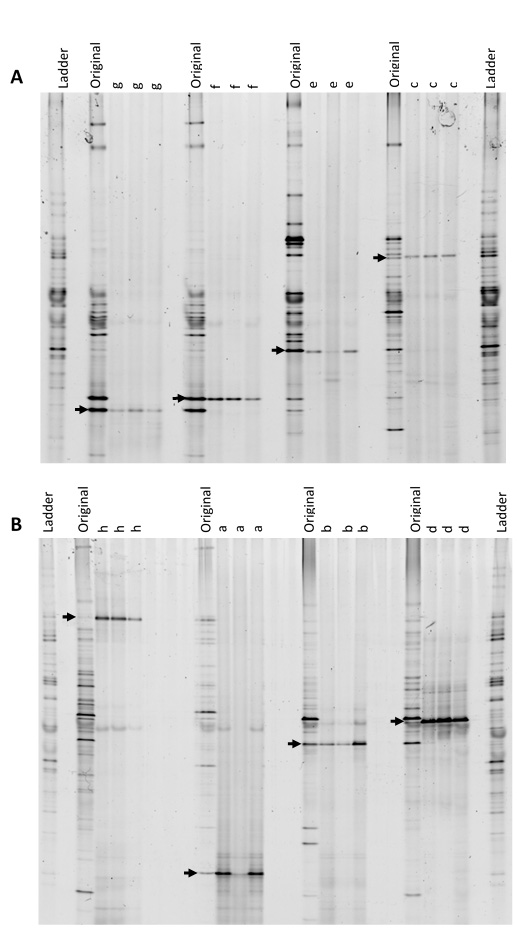


Figure S1 –DGGE to confirm selection and purity of amplicons later sequenced. Band classes (A) f, g, e and c (B) h, a, b and d ran adjacent to corresponding original sample. Arrows indicate location of band class.


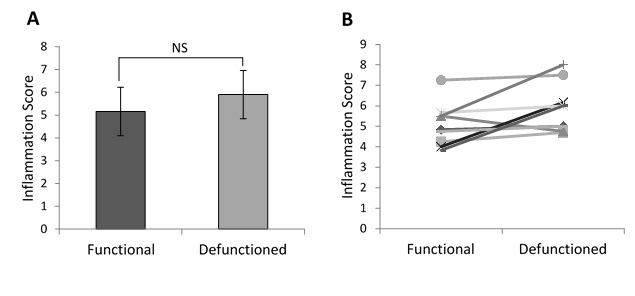
Figure S2 – (A) Average and (B) paired histological inflammation score of functional and defunctioned ileum. (n=9, p>0.05).
